# Shiga Toxin-Producing *Escherichia coli* Isolated from Wild Ruminants in Liguria, North-West Italy

**DOI:** 10.3390/pathogens13070576

**Published:** 2024-07-11

**Authors:** Valeria Listorti, Lisa Guardone, Carolina Piccinini, Isabella Martini, Carla Ferraris, Carmela Ligotti, Maria Luisa Cristina, Nicola Pussini, Monica Pitti, Elisabetta Razzuoli

**Affiliations:** 1 Istituto Zooprofilattico Sperimentale of Piemonte, Liguria and Valle d’Aosta, Via Bologna 148, 10154 Turin, Italy; isabella.martini@izsto.it (I.M.); carla.ferraris@izsto.it (C.F.); carmela.ligotti@izsto.it (C.L.); nicola.pussini@izsto.it (N.P.); monica.pitti@izsto.it (M.P.); elisabetta.razzuoli@izsto.it (E.R.); 2 Department of Veterinary Sciences, University of Pisa, Viale Delle Piagge 2, 56124 Pisa, Italy; 3 Department of Health Sciences, University of Genova, 16132 Genova, Italy; carolina.piccinini@libero.it (C.P.); cristinaml@unige.it (M.L.C.); 4 Hospital Hygiene, E. O. Galliera Hospital, 16128 Genova, Italy

**Keywords:** STEC, AMR, non-O157-STEC, roe deer, fallow deer, red deer, chamois

## Abstract

Wildlife may represent an important source of infectious diseases for humans and other wild and domestic animals. Wild ruminants can harbour and transmit Shiga toxin-producing *Escherichia coli* (STEC) to humans, and some strains even carry important antimicrobial resistance. In this study, 289 livers of wild roe deer, fallow deer, red deer and chamois collected in Liguria, north-west Italy, from 2019 to 2023 were analysed. Overall, 44 STEC strains were isolated from 28 samples. The characterisation of serogroups showed the presence of O104, O113, O145 and O146 serogroups, although for 28 colonies, the serogroup could not be determined. The most prevalent Shiga toxin gene in isolated strains was *Stx2*, and more specifically the subtype *Stx2b*. The other retrieved subtypes were *Stx1a, Stx1c, Stx1d* and *Stx2g*. The isolated strains generally proved to be susceptible to the tested antimicrobials. However, multi-drug resistances against highly critical antimicrobials were found in one strain isolated from a roe deer. This study highlights the importance of wildlife monitoring in the context of a “One Health” approach.

## 1. Introduction

Shiga toxin-producing *Escherichia coli* (STEC) are an important cause of disease in humans, and infections have been associated with a wide range of human clinical illnesses, including diarrhoea, bloody diarrhoea (haemorrhagic colitis) and haemolytic uremic syndrome (HUS) [1]. The World Health Organization estimated that 2.5 million new STEC cases occur worldwide annually [2]. STEC are the leading cause of HUS in children, and most cases of it are observed in children under 4 years of age [3]. Healthy domestic ruminants, primarily cattle, are considered the main reservoir for STEC [4]. However, even wild ruminants represent a reservoir for STEC [5,6,7] and may act as a source for human infection. Indeed, most human infections are of food-borne origin, following the ingestion of contaminated undercooked meat and dairy products, fruits and vegetables and drinking and surface water, although contact with animals or manure may also play a role in infection transmission [8]. In Europe, in 2022, the number of confirmed human cases of STEC infections of food-borne origin was 7117 [9]. 

The main STEC virulence factors are Shiga toxins, encoded by *stx1* and *stx2* genes carried by bacteriophages. *Stx* is an AB5 toxin that damages the microvascular endothelial cells of the kidney, gut and brain in humans [10]. Both Shiga toxin genes present a number of variants. *Stx2* is the most heterogeneous, comprising *Stx2a*, *Stx2b*, *Stx2c*, *Stx2d*, *Stx2e*, *Stx2f* and *Stx2g* variants, while fewer variants were reported for *Stx1*: *Stx1a, Stx1c* and *Stx1d* [11]. The variants most often associated with severe disease are *Stx2a*, *Stx2c*, *Stx2d* and *Stx1a* [12]. The primary virulence factor for intestinal colonisation is intimin, which is encoded by the eae gene and is required for intimate bacterial adhesion to epithelial cells, inducing a characteristic histopathological lesion defined as “attaching and effacing” [13]. Moreover, there are more than 470 STEC serogroups, distinguished according to the characteristics of somatic (O) and flagellar (H) antigens [14]. At present, the serogroups mainly involved in disease outbreaks are O157 and the non-O157 “Big-Six” (O26, O45, O103, O111, O121 and O145) [15]. At least forty serogroups were isolated from wildlife carriers, including many of those associated with human disease [16]. Moreover, untyped strains, of yet unknown pathogenicity, are also known to occur in wild animals [6].

Several studies investigated antimicrobial resistance in STEC, mainly in strains isolated from farmed animals, highlighting the presence of resistances even against critical human antimicrobials [17]. Antimicrobial resistances were also reported in STEC isolated from wild animals, although with low prevalence values [18]. Nevertheless, wild animals are considered potential reservoirs for antimicrobic-resistant bacteria and can contribute to their dissemination across different ecosystems [19]. Because of the relevance of STEC for public health, it is critical to investigate the distribution of these pathogens and their antimicrobial resistance patterns by adopting a “One health” approach to gather more comprehensive surveillance data. This study aimed to describe the occurrence, subtypes and serogroups of STEC isolated from wild ruminants in Liguria region, north-west Italy, between 2019 and 2023, and to investigate their antimicrobial resistance features.

## 2. Materials and Methods

### 2.1. Sampling

A total of 289 liver samples of roe deer, fallow deer, red deer and chamois were included in this study. The samples were collected in the framework of annual monitoring plans on wildlife health and the food safety of derived products, involving game hunted in Liguria from 2019 to 2023. A portion of the liver of each animal was collected by the hunters, using dedicated knives and sterile bags provided by the laboratory, and were transported refrigerated to the laboratory within 4 h after collection. The hunters were trained by laboratory workers on evisceration procedures. In the laboratory, 25 g ± 1 g of liver tissue was weighted and homogenised in 225 mL ± 5 mL of buffered peptone water.

### 2.2. STEC Detection: PCR and Isolation

A protocol slightly modified from the ISO TS 13136:2012 [20] was used for STEC detection, isolation and serotyping. The PCR analyses for the detection of *stx1*, *stx2* and *eae* genes were performed according to the above-mentioned ISO (Table S1). The PCRs were considered positive when a cq lower than 35 cycles was observed on the dedicated software (Version 2.2, Bio-rad CFX Manager Industrial Diagnostic, Hercules, CA, USA). Positive (certified materials supplied by the European reference laboratory) and negative (molecular-grade water) controls were included in each PCR run. The samples that yielded positive results for *stx1* and/or *stx2* were then plated on differential solid medium Tryptone Bile X-Gluc agar (TBX), Sorbitol MacConkey agar with cefixime and tellurite (CTSMAC) and Rhamnose MacConkey agar (RMAC) to isolate the strains. The colonies compatible for colour and characteristics to *E. coli* were then re-isolated on nutrient agar plates, and then re-tested in a PCR in pools. For each sample, ten pools of five isolated colonies each were tested using the same PCR for the detection of *stx1*, *stx2* and *eae* genes. The colonies belonging to the pools which yielded positive results for the presence of at least one of the *stx* genes were then tested singularly.

### 2.3. PCR for Serogrouping

The selected single colonies that yielded positive results for at least one of the *stx* genes were then serogrouped using different protocols for the detection of the fourteen serogroups mainly associated with human infections: O157, O145, O111, O103, O26, O121, O128, O113, O104, O91, O45, O55, O80 and O146 [21,22,23,24,25,26] (Table S2). The PCRs were considered positive when a cq lower than 35 cycles was observed on the dedicated software (Version 2.2, Bio-rad CFX Manager Industrial Diagnostic, Hercules, CA, USA). Positive (certified materials supplied by the European reference laboratory) and negative (molecular-grade water) controls were included in each PCR run.

### 2.4. PCR for Stx Subtyping

The subtypes of the *stx* genes were determined in isolated colonies, in which the presence of *stx1* and/or *stx2* had been assessed. Different protocols of standard PCRs able to identify *stx1a*, *1c*, *1d*, *Stx2a*, *2b*, *2c*, *2d*, *2e*, *2f* and *2g* were used [11,27] (Table S3). The PCR was considered positive when an amplicon of the expected length was observed on the electrophoretic gel. Positive (certified materials supplied by the European reference laboratory) and negative (molecular-grade water) controls were included in each PCR run.

### 2.5. Antimicrobial Resistances Analyses

Phenotypic testing based on the determination of minimum inhibitory concentrations (MICs) to amikacin (4–128 µg/mL), ampicillin (1–32 µg/mL), azithromycin (2–64 µg/mL), cefotaxime (0.25–4 µg/mL), ceftazidime (0.25–8 µg/mL), chloramphenicol (8–64 µg/mL), ciprofloxacin (0.015–8 µg/mL), colistin (1–16 µg/mL), gentamicin (0.5–16 µg/mL), meropenem (0.03–16 µg/mL), nalidixic acid (4–64 µg/mL), sulfamethoxazole (8–512 µg/mL), tetracycline (2–32 µg/mL), tigecycline (0.25–8 µg/mL) and trimethoprim (0.25–16 µg/mL) was performed using a commercial microdilution tool (Sensititre EU Surveillance *Salmonella*/*E. coli*–EUVSEC3, Thermo Fisher Scientific, Waltham, MA, USA) following the manufacturer’s instructions. We applied the MIC interpretive resistance standards defined by the European Committee on Antimicrobial Susceptibility Testing (EUCAST) and EFSA [28,29] to define isolates of *E. coli* resistant to amikacin > 8 µg/mL, ampicillin > 8 µg/mL, azithromycin > 16 µg/mL, cefotaxime > 0.25 µg/mL, ceftazidime > 0.5 µg/mL, chloramphenicol > 16 µg/mL, ciprofloxacin > 0.06 µg/mL, colistin > 2 µg/mL, gentamicin > 2 µg/mL, meropenem > 0.125 µg/mL, nalidixic acid > 16 µg/mL, sulfamethoxazole > 64 µg/mL, tetracycline > 8 µg/mL, tigecycline > 1 µg/mL and trimethoprim > 2 µg/mL. Multidrug resistance (MDR) was defined as resistance to at least three antimicrobial classes.

## 3. Results

A total of 28 samples out of the 289 analysed were positive for the presence of *stx1* and/or *stx2* genes at PCR on at least one single colony, accounting for a percentage of positivity of 9.7%. A total of 44 STEC colonies were isolated from these 28 positive samples (Table 1). Of these colonies, seven were positive for both the *stx2* and *stx1* genes. Twenty-seven colonies were positive only for the *stx2* gene, and ten were positive only for the *stx1* gene. Moreover, 12 isolates were positive also for the *eae* gene (Table 2). The STEC isolates were then analysed for the serogroup and subtype determination. Serogroup identification was possible only for 16 isolates. Four serogroups were identified: O104, O113, O145 and O146 (Table 2). 

The subtype of *stx1* and/or *stx2* gene was identified in 36 out of 44 isolates (82.2%). For three isolates, only the *stx1* subtype was identified, although they tested positive also for *stx2*. Moreover, it was not possible to identify the *stx2* subtype for five other isolates only positive for this gene (Table 2).

Analyses for the characterisation of the antimicrobial resistance were performed for all the 44 isolates. Only in one isolate was a multi-drug resistance against eight substances found. In detail, one isolate from the liver of a roe deer hunted in 2023 in Imperia province was resistant to cefotaxime, ceftazidime, ciprofloxacin, colistin, gentamicin, meropenem, nalidixic acid and sulfamethoxazole. The other isolates were sensitive to all the tested molecules.

## 4. Discussion

In the present survey, the occurrence of STEC in hunted wild ruminants from Liguria region, in the North-west of Italy, was studied. The pathogenetic and antimicrobial resistance characteristics of the isolates were also investigated. Cultivable STEC were recovered from 9.7% of the analysed liver samples. Studies conducted in different European areas showed different prevalence values in wild ruminants, varying from 9% to 25% [7,30,31,32]. Although not dissimilar to those obtained in other studies conducted in Europe, the percentage of positivity observed in our study could be underestimated due to the matrix used for the analyses. Most of the above-mentioned studies, in fact, analysed faecal samples. In this study, the liver was chosen as an analytical matrix for two different purposes. Firstly, we aimed to assess the risk of contamination by STEC of an edible part of the animal, often consumed not fully cooked. Secondarily, since the sampling was conducted by hunters in the field, the collection of this part instead of faecal material was chosen for hygienic reasons, to avoid soiling the carcass, which was subsequently destined for human consumption. Beside the matrix effect, the prevalence value lower than other studies in Europe [7,32] could also be related to the very low presence of cattle breeding and humans in the forested sampling areas. Indeed, the Liguria region is mainly occupied by mountains and hills, with very limited plain areas and where the forest covers over 50% of the total area of the region, sustaining populations of wild boar, roe deer and red deer, the most common wild ungulates in Liguria. 

Our study confirms, as observed in other studies, that the presence of non-O157 serogroups is more common in samples isolated from wild ruminants [7,31,32,33]. We specifically investigated the presence of the 14 serogroups most frequently involved in human cases [26], and we isolated four different STEC serogroups potentially associated with human illness. O146 was found to be the main common serogroup, as was also described in other studies conducted on wild ruminants [7]. This serogroup was found in human cases [34], and it is considered an emerging serogroup in the EU, with 198 cases reported in 2022, including HUS cases [35]. It has also been isolated in a case of HUS in a new-born infected by an asymptomatic mother during labour [36]. We also detected the serogroup O104, which, in Europe, is an important but not frequent non-O157 STEC serogroup that in 2011 caused an important outbreak related to the consumption of sprouts [37]. This serogroup had already been detected in a red deer in Italy [38]. We also found the O145 serogroup that was reported from cases with acute watery diarrhoea, bloody diarrhoea, haemorrhagic colitis and HUS in Germany [39] and was previously isolated in a red deer in Spain [33]. The serogroup O113, previously reported in wild ruminants, was also found in HUS cases [38,40,41]. 

It has to be noted that, for most of the isolates (28/44), the serogroup could not be determined. This could be due to the fact that, in this study, only 14 serogroups were specifically investigated following [26]. Moreover, in recent years, in addition to STEC belonging to non-O157 serogroups, non-typed ones have also been found to be widespread in both wild and domestic animals [6,30,32,42]. Indeed, new serogroups that harbour the stx gene and have not been typed yet are emerging and may carry multi-drug resistance genes [6]. It has been hypothesised that the occurrence of emerging and untyped STEC serogroups could be influenced by habitat modifications due to changes in climatic conditions and shifts in the availability of resources by both humans and animals [6]. Further investigations to better characterise such untyped strains are needed.

Our study confirms that the most prevalent Shiga toxin gene is *stx2*, as observed in other studies on wild ungulates [7,30]. Regarding the subtypes, *stx2b* and *stx1a* were found in this study, which are among the most common Stx subtypes detected in strains from severe human infections [9]. *stx2b* was the most commonly detected, as was observed in other studies conducted on wild ruminants [7,30,38,43]. We also detected the subtypes *stx1c*, *stx1d* and *stx2g*, which are less frequently involved in severe human infections and had also been isolated by other authors [7]. In particular, we isolated *stx2g*, which is a rare human subtype, usually reported from livestock and wild ruminants, food or waste- or surface water, and which has also been reported in patients with clinical symptoms [44]. 

In this study, an overall low prevalence of antimicrobial resistances was observed. This might reflect not only the low level of exposure of the studied wildlife species to antimicrobials, but also the low level of resistant bacteria in the areas where these animals live and feed [45]. In fact, as mentioned above, in the study area, there is a low presence of livestock and of human settlements. Indeed, another recent study conducted in the same area on wild boars revealed a low presence of antimicrobial resistances in this more anthropized species as well [46]. In several studies conducted on STEC isolated from wild ruminants, a high susceptibility of the isolates against the tested molecules was observed, and no resistances against highly critical antimicrobials were observed [30,32,33,43]. However, the presence in a single strain of significant resistances against third-generation cephalosporins, quinolones, colistin and meropenem, highlights that the role of these animals as a possible reservoir and for the transmission of important antimicrobial resistance should not be disregarded.

## 5. Conclusions

Despite the low presence of livestock in the area and the low level of urbanisation, the investigated wild ruminants resulted as carriers of STEC, and, in one case, also of important AMR. This evidence highlights the relevant role that wild ruminants could have in the dissemination of this important zoonotic pathogen and the risk to hunters, people consuming meat and meat products and others in contact with their faeces. In the future, it might be interesting to continue such monitoring, and it would be also useful to regulate STEC analyses foreseen for hunted wild ruminants and report them to facilitate possible epidemiological links with human cases. In such cases other diagnostic techniques, such as whole genome sequencing, should also be used to better characterise the circulating strains.

## Figures and Tables

**Table 1:** Liver samples analysed per year, samples tested positive to *stx1* and/or *stx2* and number of isolates which were submitted to serotyping and subtyping.

Year of Sampling	Number of Liver Samples Analysed Per Year	Samples Positive to *stx1* and/or *stx2* Genes	% of Positivity	Isolates
2019–2020	40	8	20.0%	13
2021–2022	136	15	11.0%	21
2022–2023	113	5	4.4%	10
**Total**	**289**	**28**	**9.7%**	**44**

**Table 2:** Analysed samples and isolates, with species of origin, serogroup, *stx* and *eae* genes presence and *stx* subtype.

Sample Number	Species	Isolate Number	Serogroup	*stx* and *eae* Genes	*stx* Subtype
1	roe deer	1	O146	*stx2*	*stx2b*
		2	O146	*stx2*	*stx2b*
2	roe deer	3	O146	*stx2*, *eae*	*stx2b*
3	roe deer	4	-	*stx2*, *eae*	
4	roe deer	5	-	*stx1*, *stx2*, *eae*	*stx1a*
		6	-	*stx1*, *stx2*, *eae*	*stx1a*
		7	-	*stx1*, *stx2*	*stx1a*
5	roe deer	8	-	*stx1*	*stx1a*
		9	-	*stx1*	*stx1a*
6	roe deer	10	-	*stx2*, *eae*	
7	roe deer	11	-	*stx1, eae*	*stx1d*
8	roe deer	12	-	*stx1*	*stx1d*
	roe deer	13	-	*stx1*, *eae*	*stx1d*
9	roe deer	14	-	*stx1*, *stx2*	*stx1d*, *stx2b*
10	roe deer	15	-	*stx2*	*stx2b*
11	roe deer	16	-	*stx2*	*stx2b*
12	roe deer	17	-	*stx1*	*stx1d*
13	roe deer	18	O146	*stx2*	*stx2b*
		19	O146	*stx2*	
14	roe deer	20	O146	*stx2*	
		21	O146	*stx2*	
15	fallow deer	22	-	*stx2*	*stx2b*
16	fallow deer	23	O145	*stx1*	*stx1d*
17	fallow deer	24	O113	*stx1*, *stx2*	*stx1d*, *stx2b*
		25	O113	*stx1*, *stx2*	*stx1d*, *stx2b*
18	red deer	26	-	*stx2*	*stx2g*
		27	-	*stx2*,	*stx2g*
19	fallow deer	28	-	*stx1*	*stx1d*
		29	-	*stx1*	*stx1c*
20	chamois	30	O146	*stx2*, *eae*	*stx2b*
21	fallow deer	31	O146	*stx2*	*stx2b*
22	roe deer	32	O104	*stx1*	*stx1c*
23	roe deer	33	O113	*stx2*	*stx2g*
		34	O104	*stx2*	*stx2g*
24	fallow deer	35	-	*stx1*, *stx2*	*stx1c*, *stx2b*
25	chamois	36	-	*stx2*	*stx2b*
		37	-	*stx2*	*stx2b*
		38	-	*stx2*	*stx2b*
26	chamois	39	-	*stx2*	*stx2b*
		40	-	*stx2*	*stx2b*
27	chamois	41	-	*stx2*, *eae*	*stx2b*
		42	-	*stx2*, *eae*	*stx2b*
28	roe deer	43	O104	*stx2*, *eae*	*stx2b*
		44	-	*stx2*, *eae*	*stx2b*

## Data Availability

The original contributions presented in this study are included in the article/Supplementary Material, and further inquiries can be directed to the corresponding authors.

## Supplementary Materials

The following supporting information can be downloaded at https://www.mdpi.com/article/10.3390/pathogens13070576/s1. Table S1: Primers used for the detection of *stx1*, *stx2* and *eae* genes. Table S2: Primers and probes used for serogroup characterisation. Table S3: Primer used for *stx1a/c/d* and *stx2a/b/c/d/e/f/g* gene detection. References [21,23,24,25,26,47,48] are cited in Supplementary Materials.
