# Supplementary material for: Shiga Toxin-Producing Escherichia coli Isolated from Wild Ruminants in Liguria, North-West Italy
Source: Pathogens. 2024 Jul 11;13(7):576. doi: 10.3390/pathogens13070576 (PMC11279605; doi:10.3390/pathogens13070576)
Supplement: Supplementary file 1 [file pathogens-13-00576-s001.zip › pathogens-3062483-supplementary.pdf]

Table S1: Primers used for the detection of *stx1, stx2* ed *eae* genes.

Target gene (Reference)	Primer name	Sequence
**eae** (Paton & Paton, 1998)	eaeAF	GACCCGGCACAAGCATAAGC
	eaeAR	CCACCTGCAGCAACAAGAGG
**stx1** (Paton & Paton, 1998)	stx1F	ATAAATCGCCATTCGTTGACTAC
	stx1R	AGAACGCCCACTGAGATCATC
**stx2** (Paton & Paton, 1998)	stx2F	GGCACTGTCTGAAACTGCTCC
	stx2R	TCGCCAGTTATCTGACATTCTG
**stx2f** (Schmidt et al. 2000)	128-1	AGA TTG GGC GTC ATT CAC TGG TTG
	128-1	TAC TTT AAT GGC CGC CCT GTC TCC

Table S2: Primers and probes used for serogroup characterization.

Target gene (Reference)	Primers and probe sequences
wzxO26 (Perelle *et al.*, 2004)	**FWD:** CGCGACGGCAGAGAAAATT<br>**REV:** AGCAGGCTTTTATATTCTCCAACTTT<br>**Probe:** CCCCGTTAAATCAATACTATTTCACGAGGTTGA
wzxO45 (USDA, 2019)	**FWD:** CGTTGTGCATGGTGGCAT<br>**REV:** TGGCCAAACCAACTATGAACTG<br>**Probe:** ATTTTTTGCTGCAAGTGGGCTGTCCA
wzxO55 (EURL-VTEC, 2020)	**FWD:** AATTAACGAACATAACACCCAACC<br>**REV:** ATATCTCTTCGTTACTGTGTGTATTTC<br>**Probe:** ACCTCCCGCTAAAACCCCAACTCTAGTAG
wzyO80 (EURL-VTEC, 2020)	**FWD:** TGAGAGCCAAGATCCAAGCA<br>**REV:** TGGGCCATATTCGAAGTTTGAA<br>**Probe:** TCCCAAGATTCCACGTTGAT
wzyO91 (Perelle *et al.*, 2004)	**FWD:** CGATTTTCTGGAATGCTTGATG<br>**REV: C**AATACATAGTTTGATTTGTGTTTAAAGTTTAAT<br>**Probe:** CCTGGGTTGTTAGGAACAATTTCAGCACTTC
wzxO103 (Perelle *et al.*, 2005)	**FWD:** CAAGGTGATTACGAAAATGCATGT<br>**REV:** GAAAAAAGCACCCCCGTACTTAT<br>**Probe:** CATAGCCTGTTGTTTTAT
wzxO104 (Bugarel *et al.*, 2010)	**FWD:** TGTCGCGCAAAGAATTTCAAC<br>**REV:** AAAATCCTTTAAACTATACGCCC<br>**Probe:** TTGGTTTTTTTGTATTAGCAATAAGTGGTGTC
wbdIO111 (Perelle *et al.*, 2004)	**FWD:** CGAGGCAACACATTATATAGTGCTTT<br>**REV:** TTTTTGAATAGTTATGAACATCTTGTTTAGC<br>**Probe:** TTGAATCTCCCAGATGATCAACATCGTGAA
wzyO113 (Perelle *et al.*, 2004)	**FWD:** GAGCGTTTCTGACATATGGAGTGA<br>**REV:** TTGCTATAAATGGAAGCCATTCTTT

	**Probe:** TGCATGAAATGTTTAAATGCAGCGGGT
wzxO121<br>**(USDA, 2019)**	**FWD:** AGGCGCTGTTTGGTCTCTTAGA<br>**REV:** GAACCGAAATGATGGGTGCT<br>**Probe:** CGCTATCATGGCGGGACAATGACAGTGC
wzxO128<br>**(Lin *et al.*, 2011)**	**FWD:** TCGATCGTCTTGTTCAGGTT<br>**REV:** GAATGCAATGGGCAATTAAC<br>**Probe:** GGGTTGCACAATTGGCCTCC
wzxO145<br>**(USDA, 2019)**	**FWD:** AAA CTG GGA TTG GAC GTG G<br>**REV:** CCC AAA ACT TCT AGG CCC G<br>**Probe:** TGC TAA TTG CAG CCC TTG CAC TAC GAG GC
wzyO146<br>**(EURL-VTEC 2020)**	**FWD:** ACATTCGGCGTTTTTATCTCGT<br>**REV:** GGTCAAATCTCGTGCCCATAGA<br>**Probe:** AATTTCAAGGTGCCAACTTTTCA
rfbEO157<br>**(Perelle *et al.*, 2004)**	**FWD:** TTTCACACTTATTGGATGGTCTCAA<br>**REV:** CGATGAGTTTATCTGCAAGGTGAT<br>**Probe:** AGGACCGCAGAGGAAAGAGAGGAATTAAGG

Table S3. Primer used for *stx1a/c/d* and *stx2a/b/c/d/e/f/g* genes detection. All reported from EURL-VTEC Available online: https://www.iss.it/documents/20126/0/EURL-VTEC_Method_11_Rev_1.pdf/36a945da-ce05-1cab-df19-637f8169be3d?t=1644309311442 (Accessed 30/05/024).

Gene target	Primer name	Sequence
**stx 1a**	stx1a-F1<br>stx1a-R2	CCTTTCCAGGTACAACAGCGGTT<br>GGAAACTCATCAGATGCCATTCTGG
**stx 1c**	stx1c-F1<br>stx1c-R1	CCTTTCCTGGTACAACTGCGGTT<br>CAAGTGTTGTACGAAATCCCCTCTGA
**stx 1d**	stx1d-F1<br>stx1d-R2	CAGTTAATGCGATTGCTAAGGAGTTTACC<br>CTCTTCCTCTGGTTCTAACCCCATGATA
**stx2a**	stx2a-F2<br>stx2a-R3<br>stx2a-R2	GCGATACTG**R**G**B**ACTGTGGCC<br>CCG**K**CAACCTTCACTGTAAATGTG<br>GGCCACCTTCACTGTGAATGTG
**stx 2b**	stx2b-F1<br>stx2b-R1	AAATATGAAGAAGATATTTGTAGCGGC<br>CAGCAAATCCTGAACCTGACG
**stx 2c**	stx2c-F1<br>stx2c-R2	GAAAGTCACAGTTTTTATATACAACGGGTA<br>CCGGCCAC**Y**TTTACTGTGAATGTA
**stx 2d**	stx2d-F1<br>stx2d-R1<br>stx2d-R2	AAARTCACAGTCTTTATATACAACGGGTG<br>TT**Y**CCGGCCACTTTTACTGTG<br>GCCTGATGCACAGGTACTGGAC
**stx 2e**	stx2e-F1<br>stx2e-R2	CGGAGTATCGGGGAGAGGC<br>CTTCCTGACACCTTCACAGTAAAGGT
**stx 2f**	stx2f-F1<br>stx2f-R1	TGGGCGTCATTCACTGGTTG TAATGGCCGCCCTGTCTCC

**stx 2g**	stx2g-F1	CACCGGGTAGTTATATTTCTGTGGATATC
	stx2g-R1	GATGGCAATTCAGAATAACCGCT

References

Bugarel, M., Beutin, L., Martin, A., Gill, A., Fach P. Micro-array for the identification of Shiga toxin-producing *Escherichia coli* (STEC) seropathotypes associated with Hemorrhagic Colitis and Hemolytic Uremic Syndrome in humans. *Int. J. Food. Microbiol*. **2010**, *142*, 318-29.

EURL-VTEC **2020** https://www.iss.it/documents/20126/0/EURL-VTEC_Method_11_Rev_1.pdf/36a945da-ce05-1cab-df19-637f8169be3d?t=1644309311442 (Accessed 30/05/2024)

Lin, A., Sultan, O., Lau, H.K., Wong, E., Hartman, G., Lauzon, C.R. O serogroup specific real time PCR assays for the detection and identification of nine clinically relevant nonO157 STECs. *Food Microbiol.* **2011**, *28*, 478-83.

Paton, J.C., Paton, A.W. Pathogenesis and diagnosis of Shiga toxin-producing *Escherichia coli* infections. *Clin. Microbiol. Rev.* **1998**, *11*(3), 450-479.

Perelle, S., Dilasser, F., Grout, J., Fach, P. Detection by 5'-nuclease PCR of Shiga-toxin producing *Escherichia coli* O26, O55, O91, O103, O111, O113, O145 and O157:H7, associated with the world's most frequent clinical cases. *Mol. Cell. Probes*. **2004**, *18*, 185- 92.

Schmidt, H., Scheef, J., Morabito, S., Caprioli, A., Wieler, L.H., Karch, H. A new Shiga toxin 2 variant (*Stx2f*) from *Escherichia coli* isolated from pigeons. *Appl. Environ. Microbiol.* **2000**, *66*(3), 1205-1208.

USDA, United States Department of Agriculture. Primer and Probe Sequences and Reagent Concentrations for non-O157 Shiga Toxin-Producing Escherichia coli (STEC) Real-Time PCR Assay. **2019**, MLG 5C Appendix 4, https://www.fsis.usda.gov/sites/default/files/media_file/2021-03/mlg-5-appendix-4.pdf (Accessed January 10, 2024)
